# Protective Cytomegalovirus (CMV)-Specific T-Cell Immunity Is Frequent in Kidney Transplant Patients without Serum Anti-CMV Antibodies

**DOI:** 10.3389/fimmu.2017.01137

**Published:** 2017-09-12

**Authors:** Nicolle H. R. Litjens, Ling Huang, Burç Dedeoglu, Ruud W. J. Meijers, Jaap Kwekkeboom, Michiel G. H. Betjes

**Affiliations:** ^1^Department of Internal Medicine, Nephrology and Transplantation, Erasmus MC, University Medical Center Rotterdam, Rotterdam, Netherlands; ^2^Department of Gastroenterology and Hepatology, Erasmus MC, University Medical Center Rotterdam, Rotterdam, Netherlands

**Keywords:** cytomegalovirus, T cells, B cells, cellular immunity, humoral immunity

## Abstract

The absence of anti-cytomegalovirus (CMV) immunoglobulin G (IgG) is used to classify pretransplant patients as naïve for CMV infection (CMV^neg^ patients). This study assessed whether pretransplant CMV-specific T-cell immunity exists in CMV^neg^ patients and whether it protects against CMV infection after kidney transplantation. The results show that CMV-specific CD137^+^IFNγ^+^CD4^+^ and CD137^+^IFNγ^+^CD8^+^ memory T cells were present in 46 and 39% of CMV^neg^ patients (*n* = 28) although at much lower frequencies compared to CMV^pos^ patients (median 0.01 versus 0.58% for CD4^+^ and 0.05 versus 0.64% for CD8^+^ T cells) with a less differentiated CD28-expressing phenotype. In line with these data, CMV-specific proliferative CD4^+^ and CD8^+^ T cells were observed in CMV^neg^ patients, which significantly correlated with the frequency of CMV-specific T cells. CMV-specific IgG antibody-secreting cells (ASC) could be detected at low frequency in 36% of CMV^neg^ patients (1 versus 45 ASC/10^5^ cells in CMV^pos^ patients). CMV^neg^ patients with pretransplant CMV-specific CD137^+^IFNγ^+^CD4^+^ T cells had a lower risk to develop CMV viremia after transplantation with a CMV^pos^ donor kidney (relative risk: 0.43, *P* = 0.03). In conclusion, a solitary CMV-specific T-cell response without detectable anti-CMV antibodies is frequent and clinically relevant as it is associated with protection to CMV infection following transplantation with a kidney from a CMV^pos^ donor.

## Introduction

Kidney transplant (KT) recipients are at increased risk for infections following transplantation, one of the major threats being cytomegalovirus (CMV). A primary infection or reactivation with CMV may cause a viremia and can lead to severe CMV disease with organ involvement (1). Patients at increased risk for an infection with CMV either receive prophylactic or preemptive antiviral therapy (valganciclovir) following kidney transplantation (2). Classification of the risk for a CMV infection before KT is based on the presence of anti-CMV immunoglobulin G (IgG) in the patient in combination with the CMV serostatus of the kidney donor. Immunity to viruses, for example, CMV, is dependent on adequate help from CMV-specific CD4^+^ T cells enabling production of neutralizing antibodies by CMV-specific B-cells/plasma blasts and effective cytotoxic CD8^+^ T cell (CTL) responses (3–5). Absence of neutralizing antibodies does not necessarily imply that the antigen was not encountered before as cellular immunity might still be present. Furthermore, it is known that in end-stage renal disease (ESRD) patients protective humoral immunity is not maintained and often not achieved (6, 7). Therefore, several groups have proposed to include an evaluation of cellular immunity against CMV in the risk assessment strategy to more accurately assess sensitization before transplantation (8–10). Data are scarce as to whether the presence of limited cellular immunity protects from CMV viremia after kidney transplantation and no detailed insights into which T-cell subsets are involved in just-sufficient protection are available, as cellular immunity is mainly assessed by ELISpot (11).

T-cell differentiation can be assessed by using expression of CD45RO or CD45RA and the lymph node homing chemokine receptor CCR7 which is able to identify naive T cells and different memory T cell subsets, i.e., central memory (CM), effector memory (EM), and finally terminally differentiated EM CD45RA^+^ (EMRA) (12). This is accompanied by loss of the co-stimulatory marker CD28. Particularly, an effective CMV-specific T cell response yields many highly differentiated CD28^null^ T cells (13, 14). As we have previously shown, this T-cell response adds to immunological T-cell aging observed in the circulation of ESRD patients, resulting in a shift toward more differentiated memory T cells and enhanced telomere attrition (14, 15). Whether presence of limited cellular immunity to CMV impacts T-cell aging characteristics in ESRD patients is not known.

The aim of this study is to assess CMV-specific T-cell immunity including the differentiation stage of CMV-specific T cells and to determine its clinical relevance with respect to protection from CMV viremia following transplantation in a cohort of CMV-seronegative patients. Furthermore, the impact on T-cell aging parameters was evaluated.

## Materials and Methods

### Study Population

In a cohort of stable CMV-seronegative and, as a control, age- and gender-matched CMV-seropositive recipients of a kidney from a CMV-seropositive donor (D^+^/R^−^ and D^+^/R^+^, respectively), CMV-specific immunity was assessed before transplantation and linked to CMV viremia following KT. Most of the patients (86%) received induction therapy with basiliximab (Simulect^®^, Novartis). The standard triple immunosuppression given following transplantation consisted of tacrolimus (Prograf^®^, Astellas Pharma), mycophenolate mofetil (Cellcept^®^, Roche), and prednisolone (the first 3 months post-KT), the first given to 93% and the latter two to all of the patients. The first 6 months following transplantation, antiviral prophylaxis with valganciclovir 450 mg q.d. was given and if necessary adjusted for impairment of renal function. Patient characteristics are listed in Table 1. All patients gave written informed consent to participate in this study. The study was approved by the Medical Ethical Committee of the Erasmus MC (METC number 2010-080) and conducted in accordance with the Declaration of Helsinki and the Declaration of Istanbul.

**Table 1:** Demographic and clinical characteristics of study population.

	D^+^/R^+^ (*n* = 14)	D^+^/R^−^ (*n* = 28)	*P*-value
Age in years^a,b^	50 (19–63)	47.5 (21–76)	NS
Male	50% (7)	68% (19)	NS
Cytomegalovirus (CMV) immunoglobulin G titer (AU/mL)	47.5 (9–354)		
CMV viremia	0%	46% (13)	<0.01
Time point of CMV viremia post-kidney transplant (KT) (months)		5 (2–12)	
*Renal replacement therapy*^b^			NS
– Preemptive transplantation	43% (6)	43% (12)	
– Patients on dialysis	57% (8)	57% (16)	
*Underlying kidney disease*^b^			NS
– Nephrosclerosis/atherosclerosis/hypertension	14% (2)	25% (7)	
	36% (5)	29% (8)	
– Primary glomerulopathies	21% (3)	11% (3)	
– Diabetes		3% (1)	
– Urinary tract infections/stones		3% (1)	
– Reflux nephropathy		7% (2)	
– Polycystic kidney disease – Other/unknown	29% (4)	22% (6)	
Previous KT^b^	1	1	NS
Mismatches HLA class I^a^	2 (1–4)	2 (0–4)	NS
Mismatches HLA class II^a^	1 (0–2)	1 (0–2)	NS
*Immunosuppressive medication*			
– Basiliximab induction therapy^c^	79% (11)	89% (25)	NS
– Prednisolone^d^	100% (14)	100% (28)	NS
– MMF	100% (14)	100% (28)	NS
– Tacrolimus	93% (13)	93% (26)	NS
– Switch tacrolimus → everolimus^e^	7% (1)	7% (2)	NS
Donor age in years^a,b^	42 (27–66)	57 (29–72)	<0.01

*^a^Median (min–max)*.

*^b^At pre-KT*.

*^c^Given at day 0 and day 4 post-KT*.

*^d^Given the first 3 months post-KT*.

*^e^6 months post-KT*.

*NS, not significant*.

### Detection of CMV Viremia and CMV-Specific Antibodies

Serum IgG antibodies to CMV [expressed as arbitrary units/mL (AU/mL)] were measured with an enzyme immune assay (Biomerieux, VIDAS, Lyon, France). An outcome of ≥6 AU/mL was considered positive. Patients were monitored at a 3 months interval during the first year following transplantation with respect to presence of CMV DNA. Diagnosis of a CMV-viremic episode was based on the presence of copies [expressed in international units/mL (IU/mL)] of CMV DNA in blood and established by a quantitative polymerase chain reaction at the department of Virology at the Erasmus MC. An outcome of >50 IU/mL was indicative for a CMV viremia.

### Isolation of Peripheral Mononuclear Cells (PBMCs)

Before KT, PBMCs were isolated as described in detail before (16) from heparinized blood samples drawn from CMV-seronegative and CMV-seropositive patients and stored at 10 million PBMCs per vial at −150°C until further use.

### Detection of CMV-Specific CD137-Expressing Cytokine-Producing T Cells

Peripheral mononuclear cells of 28 CMV-seronegative and 14 CMV-seropositive patients were thawed, allowed to rest for 8 h at 37°C and stimulated (5 × 10^6^ PBMCs/mL) in RPMI-1640 containing glutamax (GibcoBRL, Paisley, Scotland) supplemented with 100 IU/mL penicillin, 100 μg/mL streptomycin, and 10% heat-inactivated pooled human serum, further referred to as standard culture medium. Stimulation was performed in polystyrene tubes (BD Pharmingen, Erembodegem, Belgium) in the presence of co-stimulation CD49d (1 µg/mL; BD) without (background) or with a mixture of overlapping peptide pools covering the whole pp65 and IE-1 protein of CMV (1 µg/mL; PepTivator-CMV pp65 and IE-1; Miltenyi Biotec GmbH, Bergisch Gladbach, Germany) and brefeldin A (Golgiplug; BD Pharmingen) for 12 h. This intracellular cytokine staining assay facilitates detailed characterization of CMV-specific CD4^+^ as well as CD8^+^ T cells as these can be identified by *de novo* expression of CD137 in combination with effector molecules (17). As a positive control, PBMC of 10 CMV-seronegative and 5 CMV-seropositive patients was stimulated with the combination of phorbol myristate acetate (PMA; 50 ng/mL; Sigma Aldrich, St. Louis, MO, USA) and ionomycin (1 µg/mL; Sigma Aldrich) and treated as described earlier.

Subsequently, a surface staining was performed to identify naive (CD45RO^−^CCR7^+^) and memory T cell subsets (12). CM T cells are CD45RO^+^CCR7^+^, effector memory (EM) CD45RO^+^CCR7^−^, and terminally differentiated effector memory (EMRA) CD45RO^−^CCR7^−^. In addition, less and more differentiated T cell subsets were also identified by CD28 (i.e., less differentiated being CD28^+^ and more differentiated, lacking CD28, referred to as CD28^null^). The following monoclonal antibodies were used: brilliant violet (BV)-510-labeled anti-CD4 (Biolegend Europe BV, Uithoorn, The Netherlands), pacific blue-labeled anti-CD45RO (Biolegend), allophycocyanin-Cy7 (APC-Cy7)-labeled anti-CD8 (BD, Erembodegem, Belgium), peridinin chlorophyll-Cy5.5 (PerCP-Cy5.5)-labeled anti-CD28 (BD), and phycoerythrin-Cy7 (PE)-Cy7-labeled anti-CCR7 (BD). Following fixation and permeabilization, cells were stained intracellular using APC-labeled anti-CD137 (BD) and PE-labeled anti-IFNγ (BD Pharmingen). IL-2-producing cells were only evaluated in a fraction of the patients tested, i.e., 12 CMV-seronegative and 6 CMV-seropositive patients by co-staining intracellular using fluorescein isothiocyanate-labeled anti-IL-2 (BD). Samples were measured on the FACSCanto II (BD Pharmingen), aiming for 0.5–1 × 10^6^ of T cells to be acquired, and analyzed using FACSDiva software version 6.1.2 (BD). The gating strategy for identifying CMV-specific CD137^+^CD4^+^T cells within the different subsets and in combination with cytokine production are shown in Figure S1 in Supplementary Material, a similar approach was followed for CD8^+^ T cells. The median (IQ range) background of CD137-expressing CD4^+^ T cells of all samples amounted to 0.05% (0.03–0.07%) whereas that of CD137-expressing CD8^+^ T cells was higher, amounting to 0.44% (0.23–1.02%). The median background value for CD137^+^IFNγ^+^CD4^+^ and CD8^+^ and CD137^+^IL-2^+^CD4^+^ T cells of all samples were 0.01% (0.01–0.02%), 0.04% (0–0.09%), and 0.01% (0.01–0.01%), respectively. Most of the background signal within CD4^+^ T cells was observed in cells co-expressing CD28 and of a CM/EM phenotype whereas that observed for CD8^+^ T cells were predominantly lacking CD28 and of the EM/EMRA phenotype. Since frequencies obtained for the various parameters differed considerably amongst patients, we subtracted the unstimulated value per patient from that after CMV-peptide stimulation to calculate the net signal as shown in the results. A positive detectable CMV-specific response was identified if the net response was over 0. Only detectable CD4^+^ and CD8^+^CD137^+^ CMV-specific T cell responses were analyzed in more detail with respect to cytokine production and phenotypic aspects.

### Detection of CMV-Specific Proliferating T Cells

Peripheral mononuclear cells of 12 CMV-seronegative and 6 CMV-seropositive patients were thawed and labeled with carboxyfluorescein diacetate succinimidyl ester according to the manufacturer’s instruction (CFSE; Molecular Probes^®^, The Netherlands) and subsequently stimulated in triplicate in standard culture medium at 5 × 10^4^/well (96 wells-round bottom-shaped plate) without or with CMV-lysate (30 µg/mL; Microbix Biosystems Inc., ON, Canada) or with a mixture of overlapping peptide pools covering the whole pp65 and IE-1 protein of CMV (both at a final concentration of 1 µg/mL; PepTivator-CMV pp65 and IE-1; Miltenyi Biotec). Stimulation with CMV-lysate might allow for characterization of the total pool of CMV-specific CD4^+^ T cells as the overlapping peptide pools cover only two (albeit dominant) peptides of the CMV protein. Proliferation of CMV-specific CD8^+^ T cells was only analyzed following stimulation with overlapping peptide pools of pp65 and IE-1 and not whole CMV lysate due to their restriction with respect to length (number of amino acids) of the peptides presented in the context of HLA class I. Following 6 days, cells were harvested, stained using monoclonal antibodies directed against CD3, CD4, CD8, and CD28 (again to identify less differentiated versus more differentiated subsets amongst proliferating T cells), and dead cells were excluded using 7-aminoactinomycin D. Samples were measured on the FACSCanto II (BD) and analyzed using FACS Diva software version 6.1.2 (BD), percentages of CMV-specific proliferating T cells were calculated by subtracting percentages of T cells proliferating in absence of these stimuli. The median (IQ range) of proliferating CD4^+^ and CD8^+^ T cells in absence of a stimulus amounted to 0.83% (0.50–2.48%) and 1.42% (0.69–3.36%), respectively. A positive detectable response was identified if the net response was over 0.

### Total and CMV-Specific IgG Antibody-Secreting Cells (ASC)

Peripheral mononuclear cells of 11 CMV-seronegative and, as a positive control, 4 CMV-seropositive patients were thawed and subsequently stimulated at a density of 2 × 10^6^/mL for 5 days with R848 and recombinant IL-2, according to the manufacturer’s instruction (U-Cytech BV, Utrecht, The Netherlands). On day 4, the wells of a 96-well plate were coated overnight at 4°C according to the manufacturer’s instruction. Coating consisted of either an antihuman IgG antibody, for enumeration of total IgG ASC or CMV lysate (30 µg/mL Microbix Biosystems Inc., ON, Canada), for enumeration of CMV-specific IgG ASC. Following stimulation, cells were harvested, counted, and added to the coated ELISpot plate at different concentrations, each in triplicate. After a 7 h incubation (37°C, 5% CO_2_), cells were lysed and debris washed away using PBS/0.05% Tween-20. Subsequently, the wells of the ELISpot plate were incubated for 1 h at 37°C with a biotinylated antihuman IgG (detection) antibody and upon washing followed by an incubation with phi-labeled anti-biotin antibody (GABA) for 1 h at 37°C. Finally ASC were visualized, upon a washing procedure, using an activation solution (U-Cytech BV) resulting in a silver precipitate upon incubation in the dark at room temperature. Color development was stopped using di-ionized water and spots were counted using an ELISpot reader (Bioreader^®^-600V, BIO-SYS GmbH, Karben, Germany). CMV-IgG ASC are expressed as number/10^5^ cells and a frequency of total IgG ASC.

In addition to analyzing ASC, a sample before and following stimulation was analyzed for B cell blast formation by flow cytometry. The following panel of monoclonal antibodies was used: BV510-labeled anti-CD19 (Biolegend), BV421-labeled anti-CD38 (BD), and PE-Cy7-labeled anti-CD27 (eBioscience). Plasma blasts were CD19^+^CD38^high^CD27^+^.

### Absolute Numbers of CD4^+^ and CD8^+^ T Cells and T-Cell Differentiation Status by Flow Cytometry

Absolute numbers of T-cell subsets (18) and T-cell differentiation status (19) were determined using a whole-blood staining as described in detail before.

### Telomere Length Assay

Relative telomere length of CD4^+^ and CD8^+^ T cells were determined by Flow fluorescent *in situ* hybridization (flow-FISH) as described previously (15, 20).

### Statistical Analyses

Statistical analyses were performed using GraphPad Prism version 5.01. Non-parametric Mann–Whitney *U*-test or Kruskal–Wallis test followed by a *post hoc* analysis (Dunns multiple comparison test). Categorical variables were compared using the Fisher’s exact test. The non-parametric Spearman rank correlation coefficient (Spearman’s rho, *R*s) was used to evaluate associations between various parameters. *P*-values <0.05 for two sides were considered statistically significant.

## Results

### Study Population Characteristics

The demographic and clinical characteristics of the study population are given in Table 1. Twenty-eight CMV-seronegative and 14 age- and gender-matched CMV-seropositive patients were included in this study. Both groups were well matched with no significant differences in clinical characteristics. Approximately half of the CMV-seronegative versus none of the CMV-seropositive patients experienced a CMV viremia within 12 months after transplantation (*P* < 0.01).

### CMV-Seronegative Patients Frequently Have CMV-Specific T Cells

**Figure 1:**
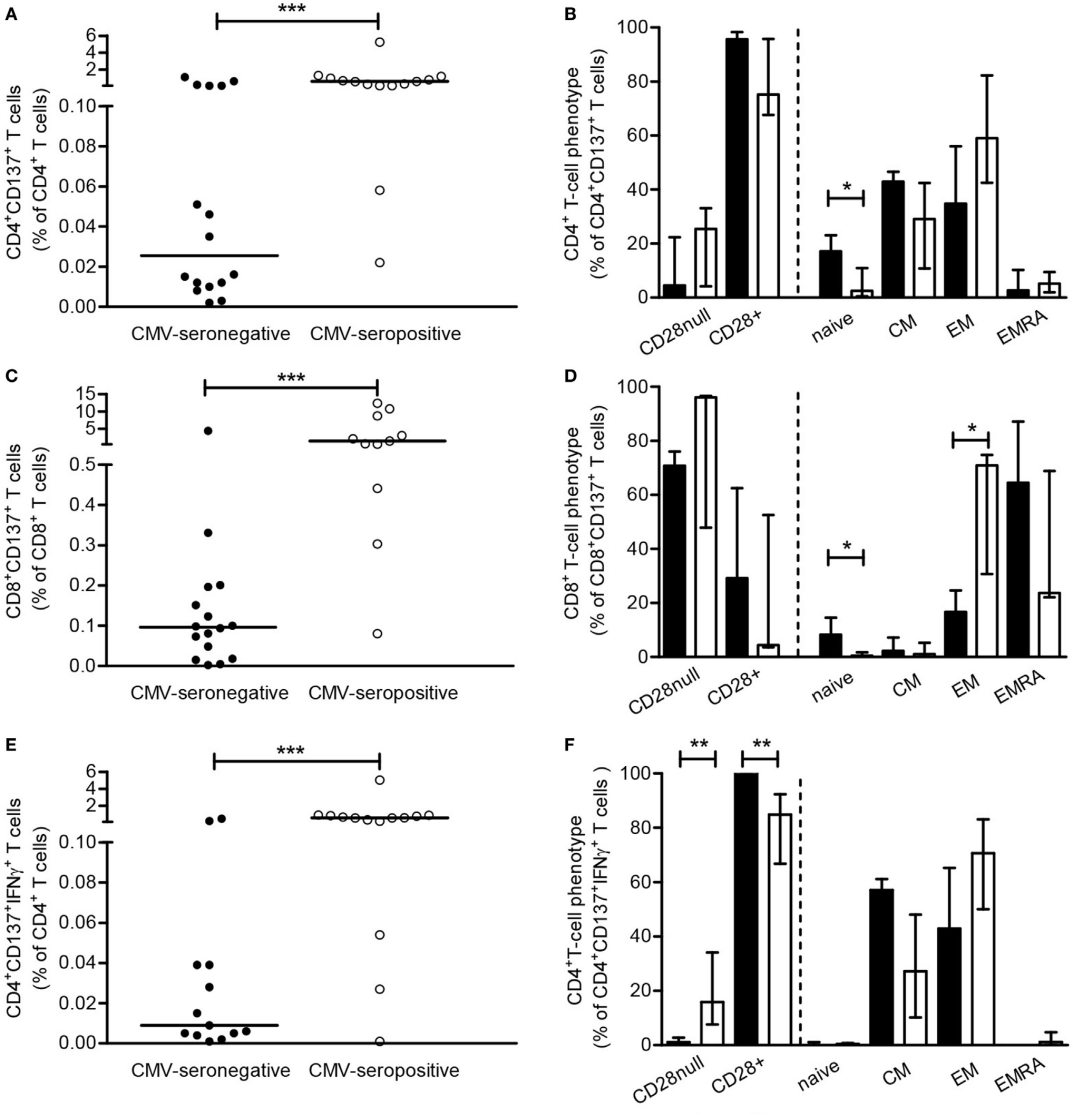

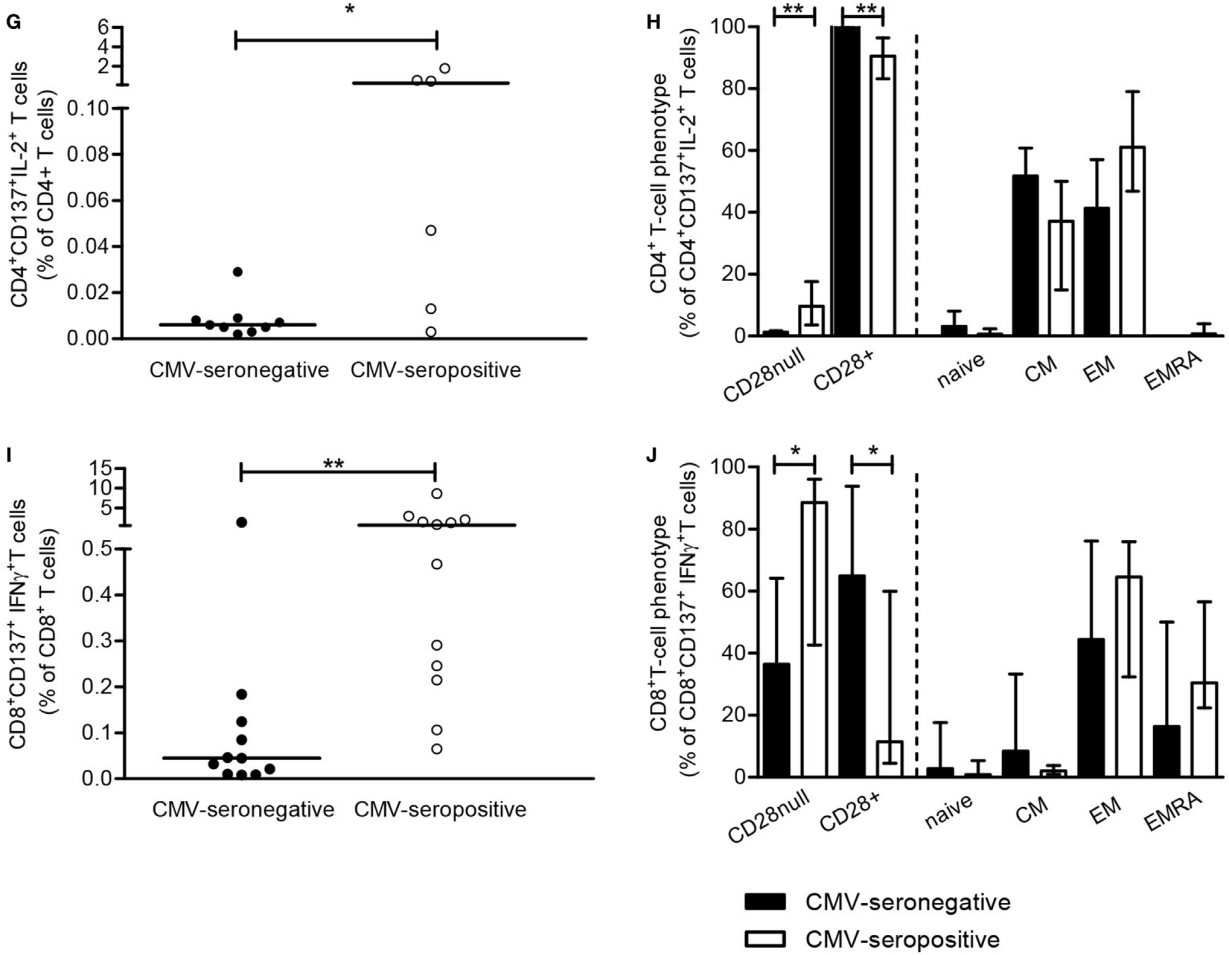
Cytomegalovirus (CMV)-specific CD137-expressing and cytokine-producing T cells. Peripheral mononuclear cells of patients were stimulated for 12-h in presence of brefeldin A and αCD49d alone or with a mixture of ppp65 and IE-1 overlapping peptide pools. Subsequently, cells are cell surface and intracellular stained to determine frequencies and phenotypic characteristics of CMV-specific CD137-expressing T cells as well as those producing cytokines. CMV-specific CD137-expressing CD4^+^ **(A)** and CD8^+^ **(C)** T cells, corrected for background (αCD49d only), are depicted as a percentage of total CD4^+^ or CD8^+^ T cells and the uncorrected values are set to 100% and dissected for CD4^+^ **(B)** or CD8^+^ **(D)** T cells co-expressing or lacking CD28 and the different naive and memory T cell subsets. A similar approach is followed for CMV-specific CD137-expressing IFN-γ and IL-2-producing CD4^+^ [**(E,G)** and dissection in **(F,H)**] and CD8^+^ [**(I)** and dissection in panel **(J)**] T cells, respectively. Closed and open symbols/bars represent CMV-seronegative (with a detectable response) and CMV-seropositive patients, respectively, and bars depict medians and interquartile range of the patient groups. *P < 0.05; **P < 0.01, ***P < 0.001.

Cytomegalovirus-specific CD4^+^ and CD8^+^ T cells, identified by expression of CD137 upon stimulation with a peptide pool of the two immunodominant proteins pp65 and IE-1 (CD137^+^CD4^+^ and CD137^+^CD8^+^) were present in 16 out of 28 CMV-seronegative patients. Median (IQ range) values of the positive responses amounted to 0.03% (0.01–0.11%) and 0.10% (0.03–0.18%) for CD4^+^ and CD8^+^ T cells, respectively (Figures 1A,C, black dots). A positive correlation was observed between the CD4^+^ and CD8^+^ level of CMV-specific T-cell response in CMV-seronegative patients (*R*s = 0.50, *P* < 0.05). The percentages of CMV-specific CD137-positive CD4^+^ and CD8^+^ T cells (present in 14 and 11 out of 14 CMV-seropositive patients, respectively) were significantly (*P* < 0.001) higher in CMV-seropositive patients and amounted to 0.61% (0.11–1.04%) and 1.45% (0.44–8.69%) (Figures 1A,C, open dots), respectively.

Cytomegalovirus-specific CD137^+^CD4^+^ and CD137^+^CD8^+^ T cells were predominantly of the memory phenotype in both CMV-seropositive as well as CMV-seronegative patients. However, significantly higher percentages (*P* < 0.05) of CD137^+^CD4^+^ and CD137^+^CD8^+^ T cells in CMV-seronegative patients had a naive phenotype when compared to CMV-seropositive patients (Figures 1B,D, respectively). No differences were observed when comparing the maximal capacity of T cells to express CD137 between CMV-seronegative and CMV-seropositive patients upon PMA/ionomycin stimulation (Figures S2A,B in Supplementary Material).

### CMV-Specific T Cells in CMV-Seronegative Patients Produce IFNγ and IL-2

Next, the presence and distribution of IFNγ- and IL-2-producing CMV-specific T cells were studied. In 13 out of 28 CMV-seronegative patients, CMV-specific IFNγ^+^CD137^+^CD4^+^T cells were present and median (IQ range) frequencies amounted to 0.01% (0.01–0.04%) versus 0.58% (0.16–0.87%) for CMV-seropositive patients, that all had CMV-specific IFNγ^+^CD137^+^CD4^+^T cells (Figure 1E, *P* < 0.001). In CMV-seronegative patients, approximately 37% of the CMV-specific CD137-expressing CD4^+^T cells produced IFNγ compared to 69% in CMV-seropositive patients (*P* < 0.05). IFNγ-producing CMV-specific CD137^+^CD4^+^ T cells all co-expressed CD28, and similar frequencies were present within the CM and EM subset of CMV-seronegative patients. In CMV-seropositive patients, a slightly more differentiated phenotype was observed as 16% (*P* < 0.01) of these cells lacked CD28 and 75% were classified as EM T cells (Figure 1F).

In 12 CMV-seronegative and 6 CMV-seropositive patients, frequencies of IL-2-producing CD4^+^ T cells specific for CMV were evaluated as we have previously shown these to be associated with anti-CMV IgG titers (6). CMV-specific CD137^+^CD4^+^IL-2^+^ T cells were detected in 9 out of 12 (75%) of CMV-seronegative versus all of the CMV-seropositive patients and median (IQ range) frequencies amounted to 0.01% (0.01–0.01%) and 0.26% (0.01–0.87%) for CMV-seronegative and CMV-seropositive patients (*P* < 0.05; Figure 1G). Forty percent of CMV-specific CD137-expressing CD4^+^ T cells of CMV-seronegative patients produced IL-2 compared to 37% in CMV-seropositive patients. In CMV-seronegative patients almost all IL-2-producing cells co-expressed CD28, in contrast to the presence of CD28^null^ CMV-specific IL-2-producing cells (10%) in the CMV-seropositive group (*P* < 0.01; Figure 1H).

IFN-γ-producing CD137^+^CD8^+^ T cells were detected in 11 out of 28 (39%) CMV-seronegative and 11 out of 14 (79%) CMV-seropositive patients. Median (IQ range) frequencies amounted to 0.05% (0.01–0.12%) and 0.64% (0.22–1.90%) within the total CD8^+^ T cell population for CMV-seronegative and CMV-seropositive patients, respectively (Figure 1I, *P* < 0.01). Only 9% of CD137-expressing CD8^+^ T cells produced IFNγ in CMV-seronegative versus 36% of CMV-seropositive patients (*P* < 0.01). The differentiation status of these cells was similar for both groups and most IFNγ^+^CD137^+^CD8^+^ T cells were found within the EM and highly differentiated EMRA subsets (Figure 1J). In CMV-seronegative patients, 65% of CMV-specific IFNγ-producing cells co-expressed CD28, in contrast to the presence of 89% CD28^null^IFNγ-producing cells in CMV-seropositive patients (*P* < 0.05; Figure 1J). The frequency of CMV-specific CD137^+^CD8^+^ T cells producing IL-2 was low, i.e., median and IQ range amounted to: 0.001% (0.001–0.001%) and 0.024% (0.01–0.05%) of the total CD8^+^ T cells for CMV-seronegative and CMV-seropositive patients, respectively. As less than 5% of CD137-expressing CD8^+^ T cells produced IL-2, no further dissection into T-cell subsets was done.

The CMV-specific CD137^+^CD4^+^ and CD8^+^ T cells with a naive phenotype, present at higher frequencies in CMV-seronegative patients (Figures 1B,D) did not produce IL-2 (Figure 1H) or IFNγ (Figures 1F,J).

Collectively, these data show that in a substantial fraction of CMV-seronegative patients cytokine-producing cells are present within CMV-specific CD137-expressing T cells, although at lower frequencies. Similar to CMV-seropositive patients, these are mainly of the memory phenotype albeit less differentiated as they are more CD28^+^. No differences were observed when comparing the maximal capacity of T cells to express CD137 (Figures S2A,B in Supplementary Material) and produce cytokines between CMV-seronegative and CMV-seropositive patients (Figures S2C–E in Supplementary Material).

### CMV-Specific T Cell Proliferation in CMV-Seronegative Patients

**Figure 2:**
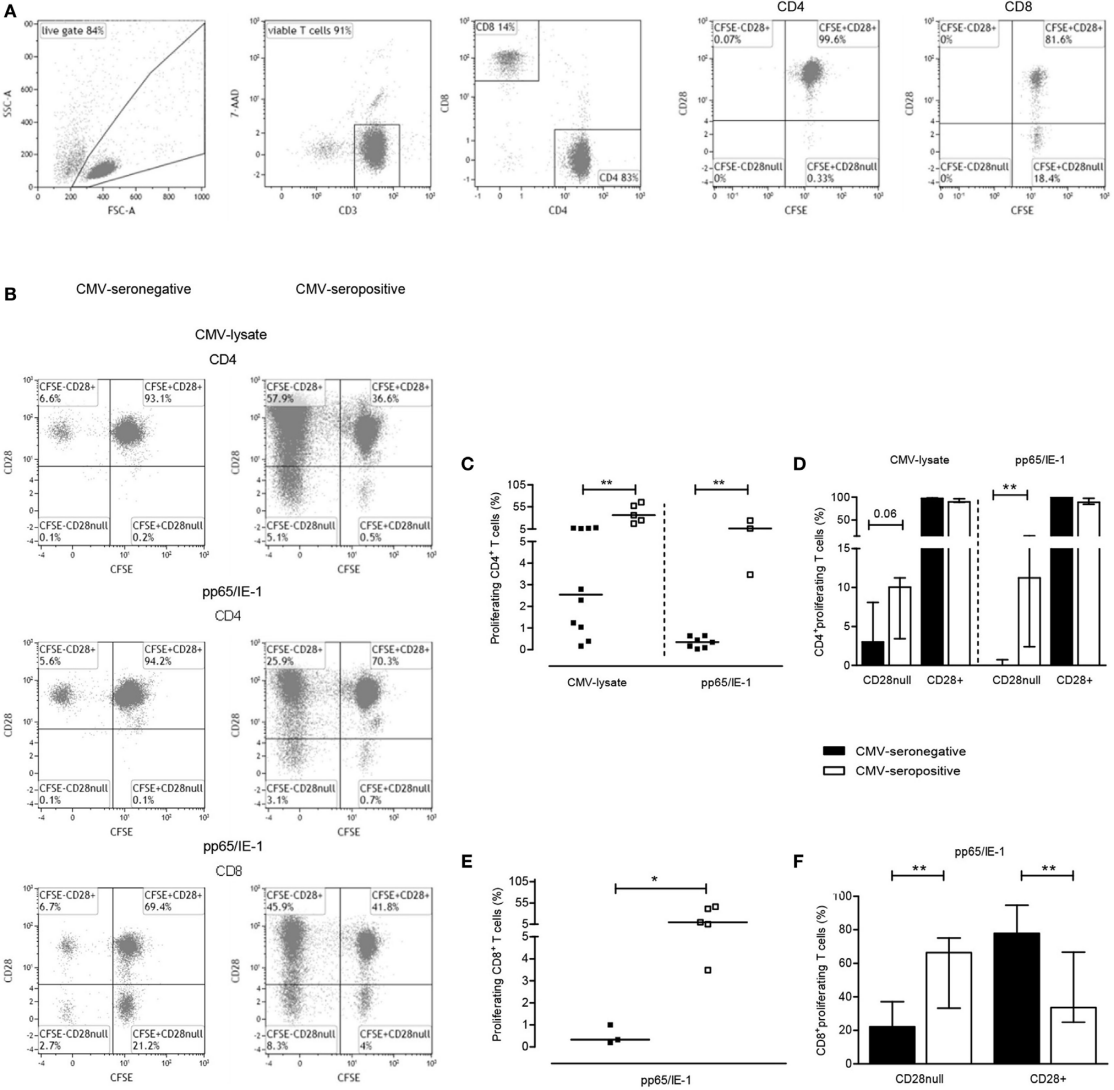
Cytomegalovirus (CMV)-specific T-cell proliferation. A typical example of the gating strategy for analysis of CMV-specific T cell proliferation is shown. Briefly, based on forward/sideward characteristics live cells are gated and depicted in a dot plot to further select living (7AAD-negative) CD3^+^ T cells **(A)**. These were then further dissected into CD4^+^ and CD8^+^ T cells **(A)**. CD4^+^ and CD8^+^ T cells are finally plotted to visualize proliferation (CFSE dilution) and CD28 expression and a typical example for background proliferation within CD4^+^ and CD8^+^ T cells is shown in the last two plots in panel A. In panel **(B)**, typical (detectable) proliferative responses of CD4^+^ and CD8^+^ T cells to CMV-lysate (CD4^+^ T cells alone; upper graphs) or a mixture of overlapping peptides for pp65/IE-1 (both CD4^+^ and CD8^+^ T cells; middle and lower graphs, respectively) are depicted for a CMV-seronegative (left panel) and CMV-seropositive patient (right panel). Percentages of CMV-specific proliferating CD4^+^ and CD8^+^ T cells, corrected for frequencies of proliferating CD4^+^ or CD8^+^ T cells in absence of a stimulus, are depicted in panels **(C,E)**, respectively. A dissection of uncorrected CMV lysate- or peptide-induced proliferating T cells (set to 100%) into those lacking or expressing CD28 are shown in panels **(D,F)** for CD4^+^ and CD8^+^ T cells, respectively. CMV-seronegative (with a detectable response) and CMV-seropositive patients are shown in closed and open symbols, respectively, and bars represent medians and interquartile range of the patient groups. *P < 0.05; **P < 0.01.

In addition to measuring the capacity to exert effector function by producing cytokines, we evaluated CMV-specific T cell proliferation by using both CMV-lysate and the peptide pool of the immunodominant proteins pp65 and IE-1 (Figures 2A,B, typical flowcytometric example). CMV-lysate-induced proliferation was observed in 10 out of 12 CMV-seronegative KT-recipients for CD4^+^ T cells, respectively. Median (IQ range) percentages of proliferating CD4^+^ T cells amounted to 2.2.55% (0.88–7.09%) (Figure 2C). Proliferation in response to the mixture of pp65- and IE-1-overlapping peptide pools was observed in a smaller proportion of CMV-seronegative patients, i.e., 7 and 3 out of 12 KT recipients for CD4^+^ and CD8^+^ T cells [median and IQ range: 0.35 and 0.09–0.64% (Figure 2C) and 0.33 and 0.19–1.00% (Figure 2E), respectively]. CD4^+^ T cells of CMV-seropositive patients proliferated vigorously to CMV-lysate. The median percentage of proliferating CD4^+^ T cells was 36% (Figure 2C). Proliferation in response to the mixture of pp65- and IE-1-overlapping peptide pools amounted to 6.34% (3.47–24.32%) and 9.64% (4.26–43.63%) for CD4^+^ and CD8^+^ T cells, respectively (Figures 2C,E). In CMV-seronegative patients, most (>97%) of the proliferating CD4^+^ T cells co-expressed CD28, indicative for a less differentiated phenotype. In CMV-seropositive patients approximately 10% of all proliferating CD4^+^ T cells were CD28^null^ (Figure 2D). This difference in differentiation status was even more pronounced in proliferating CD8^+^ T cells as 78% of these cells in CMV-seronegative patients expressed CD28 compared to approximately 34% of CMV-seropositive patients (*P* < 0.05) (Figure 2F). Frequencies of CMV-specific IFNγ-producing CD137^+^CD4^+^ T cells and IL-2-producing CD137^+^CD4^+^ T cells were positively correlated with percentages of proliferating CD4^+^ T cells [for IFNγ: *R*s = 0.63; *P* = 0.02 (CMV-lysate) and *R*s = 0.71; *P* = 0.02 (pp65/IE-1 peptides), for IL-2: *R*s = 0.62; *P* = 0.02 (CMV-lysate) and *R*s = 0.74; *P* = 0.01 (pp65/IE-1 peptides)].

### Low Frequency of CMV-Specific IgG ASC in CMV-Seronegative Patients

**Figure 3:**
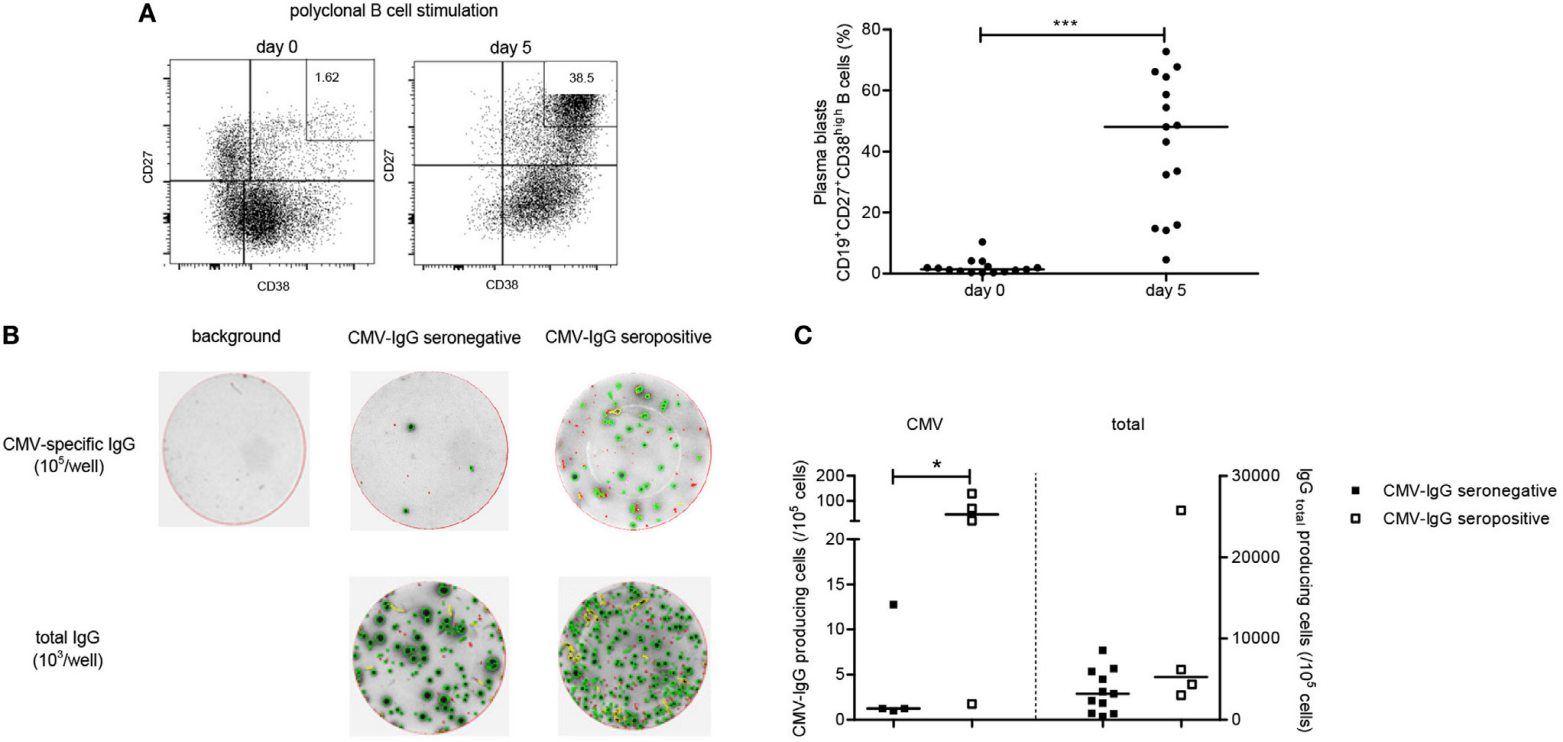
Cytomegalovirus (CMV)-specific B-cell responses. A B cell ELISpot assay was performed to enumerate CMV-specific and total immunoglobulin G (IgG) antibody-secreting cells (ASC). In panel **(A)**, a typical flow cytometric example is given of B cell blasts, identified as CD19^+^CD27^+^CD38^high^ cells, before a following a 5-day stimulation with R848 and recombinant IL-2 on the left side. On the right side, the individual and median frequencies of B cell blasts are depicted before stimulation and following a 5-day stimulation for 15 kidney transplant recipients (11 CMV-seronegative and 4 CMV-seropositive patients). Following B-cell stimulation, cells were transferred at different numbers/well to wells of a 96-well B-ELISpot plate coated with CMV-lysate or an antihuman IgG antibody or not coated (containing PBS; background IgG-producing cells). In panel **(B)**, representative examples are shown for background, CMV-specific and total IgG ASC as determined within CMV-seropositive and CMV-seronegative patients. In panel **(C)**, individual and median (detectable) CMV-specific and total IgG ASC, corrected for background (number of spots in absence of a coating antibody) for CMV-seropositive (open symbols) and CMV-seronegative (closed symbols) patients are depicted. *P < 0.05; ***P < 0.001.

Next we evaluated whether CMV-specific memory B cells could be detected in the circulation of CMV-seronegative patients. The frequency of plasma blasts within the circulation is very low but can be induced from B cells upon polyclonal stimulation. The total B cell number and responses may be negatively affected in ESRD patients and therefore we first documented that this protocol can indeed induce plasma blasts. As shown in Figure 3A, plasma blasts were induced (CD27^+^CD38^high^CD19^+^, from 1.4 to 48.1%) following a 5-day polyclonal stimulation. Next, total IgG and CMV-IgG ASC were quantified by ELISpot. CMV-IgG ASC were detected above background in 4 out of 11 (36%) of CMV-seronegative patients and net median (IQ range) CMV-specific IgG ASC amounted to only 1/10^5^ (1–10/10^5^), i.e., 0.08% (0.02–0.14%) of total IgG ASC (Figures 3B,C). All CMV-seropositive patients had detectable CMV-IgG ASC [median 45 cells/10^5^ (7–114/10^5^), 0.38% (0.11–1.70%) of total IgG ASC]. Interestingly, CMV-specific IFNγ- as well as IL-2-producing CD137^+^CD4^+^ T cells of the total patient group (CMV-seropositive and CMV-seronegative patients) were positively correlated with numbers of CMV-specific IgG ASC (*R*s = 0.52; *P* < 0.05 and *R*s = 0.53; *P* < 0.05, respectively).

### CMV-Specific CD137^+^IFNγ^+^CD4^+^ T Cell Responses Protect against CMV Viremia

The CMV-specific T-cell response in CMV-seronegative patients was evaluated for clinical relevance by relating it to the occurrence of CMV viremia after transplantation. In 13 out of 28 (46%) CMV-seronegative patients, a CMV viremia developed during the first year following transplantation with a CMV-seropositive donor kidney. CMV-seronegative patients with CMV-specific CD137^+^IFNγ^+^CD4^+^ T cells before transplantation had a lower risk to develop a CMV viremia after transplantation with a CMV-seropositive donor kidney compared to those without (relative risk: 0.43, *P* = 0.03). No differences in percentages of CMV-specific IFNγ-producing CD137^+^CD4^+^ T cells were observed between CMV-seronegative patients without and those with a CMV viremia (data not shown).

### CMV-Associated T-Cell Defects Mimicking T-Cell Aging Are Not Present in CMV-Seronegative Patients with CMV-Specific T Cells

Cytomegalovirus latency leaves a clear fingerprint on the T cell immune system. The CMV-associated changes in the circulating T cell compartment of CMV-seropositive ESRD patients comprise of a reduced telomere length, significant expansion of the CD8^+^ T cell pool and increased differentiation status of the memory T cells, notably the induction of CD28^null^ T cells in both CD4^+^ and CD8^+^ T cells (21, 22). However, such changes were not observed when comparing CMV-seronegative patients with and without detectable CMV-specific CD137-expressing IFNγ-producing CD4^+^ T cells (Table 2).

**Table 2:** Effects of CMV-immunity on T-cell parameters before transplantation.

	CMV^pos^ ESRD patients (*N* = 14)	CMV^neg^ ESRD patients without CMV-specific CD137^+^IFNγ^+^CD4^+^ T cells (*N* = 15)	CMV^neg^ ESRD patients with CMV-specific CD137^+^IFNγ^+^CD4^+^ T cells (*N* = 13)	*P*-value*
T cells	1,184 (887–1,519)	1,039 (636–1,360)	1,057 (750–1,340)	ns
CD4^+^ T cells	629 (444–786)	714 (373–916)	639 (451–821)	ns
CD4^+^ T_naive_ cells	110 (73–173)	213 (119–531)	226 (163–354)	ns
CD4^+^ T_MEM_ cells	535 (292–641)	385 (185–512)	434 (255–641)	ns
CD4^+^ T_CM_ cells	263 (174–389)	227 (137–366)	283 (176–363)	ns
CD4^+^ T_EM_ cells	185 (106–301)	116 (100–148)	108 (80–273)	ns
CD4^+^ T_EMRA_ cells	7 (5–21)	11 (4–18)	8 (3–13)	ns
CD4^+^CD28^null^ T cells	29 (3–65)	3 (2–5)	3 (1–20)	ns
CD4^+^CD31^+^ T_naive_ cells	63 (43–123)	170 (61–264)	144 (91–222)	ns
CD8^+^ T cells	465 (316–618)	257 (168–445)	240 (213–469)	ns
CD8^+^ T_naive_ cells	84 (39–120)	104 (59–198)	84 (44–179)	ns
CD8^+^ T_MEM_ cells	349 (271–528)	140 (87–277)	191 (167–246)	ns
CD8^+^ T_CM_ cells	15 (9–88)	16 (7–30)	25 (13–44)	ns
CD8^+^ T_EM_ cells	110 (59–144)	85 (46–188)	83 (53–122)	ns
CD8^+^ T_EMRA_ cells	151 (85–287)	37 (25–69)	41 (18–76)	ns
CD8^+^CD28^null^ T cells	228 (85–276)	37 (24–86)	58 (23–67)	ns
CD8^+^CD31^+^ T_naive_ cells	79 (39–118)	98 (57–175)	76 (41–175)	ns
CD4^+^ RTL	10.20 (9.2–15.2)	11.2 (9.1–17.8)	12.1 (9.4–14.1)	ns
CD8^+^ RTL	10.85 (7.7–14)	13 (9.7–21.2)	11.4 (9.1–16)	ns

*Cell numbers are given in cells/μL blood; data represent median (interquartile range)*.

*When comparing CMV^neg^ ESRD patients without to those with CMV-specific CD137^+^IFNγ^+^CD4^+^ T cells.

*MEM, memory; CM, central memory; EM, effector memory; EMRA, terminally differentiated effector memory (CD45RA^+^); RTL, relative telomere length given as a %; ESRD, end-stage renal disease*.

## Discussion

In this study, we evaluated the presence of CMV-specific cellular and humoral immunity in a cohort of CMV-seronegative patients and assessed the clinical relevance with respect to the risk for a CMV viremia after kidney transplantation. Data revealed the presence of a low frequency of CMV-specific T-cells in over half of these patients. Interestingly, in particular IFNγ-producing CMV-specific CD4^+^ T cells were associated with protection from CMV viremia following transplantation with a kidney from a CMV-seropositive donor. Thus, a protective anti-CMV cellular immunity may exist in the absence of serum anti-CMV IgG.

Presence of cellular immunity in the absence of protective antibodies is not uncommon. Possible explanations for this disassociation may be that either cellular immunity is inefficient to induce adequate humoral immunity (6, 7) or that protective antibodies are not adequately maintained. Both these mechanisms may hold true for patients with ESRD. It is known that T-cell dependent vaccines (e.g., HBsAg, tetanus toxoid, and diphtheria) are poor inducers of protective antibody titers in these patients, and antibody titers are poorly maintained (23–25). A detailed analysis of the dynamics and quality of the antigen-specific T cell response after vaccination with HBsAg in ESRD patients showed a poor generation of memory CD4^+^ T cells (6, 7). In particular, the presence of antigen-specific CD4^+^ EM T cells correlated significantly with the titers of anti-HBsAg and a similar relation could be shown for anti-CMV IgG. The results of this study also showed a statistically significant relation between the presence of IL-2 or IFNγ-producing CMV-specific CD4^+^ T cells and anti-CMV IgG ASC. Therefore, a weak induction of a CMV-specific T cell response may underlie the inadequate generation of an anti-CMV humoral response. In addition, it could still be possible that a low but detectable anti-CMV humoral response was generated initially but not adequately maintained.

Although there may be concerns associated with reliably measuring low frequencies of CMV-specific T cells, the multi-parameter CD137 flow cytometric assay as described previously (17), allows for a detailed characterization of total pool of antigen-specific T cells which is related with other parameters analyzed within this paper and is associated with clinical outcome, i.e., CMV viremia. The detailed analysis of the CMV-specific response within T cell subsets revealed that CMV-specific memory CD4^+^ and CD8^+^ T cells were less differentiated in the CMV-seronegative patients than in CMV-positive patients. This was evident by relatively more CD28^+^ memory T cells and less CMV-specific T cells producing effector cytokines such as IFNγ and IL-2. Of interest was the detection of a CMV-specific CD137^+^ response in phenotypically naive T cells that did not produce cytokines. These cells may be part of so-called early branched off memory T cells that are phenotypically naive T cells but are in fact memory T cells in an early developmental stage of which some are able to exert effector function (26). Again this finding supports the concept of a less well-differentiated CMV-specific T-cell response. In this respect, a paper by Redeker et al. (27) nicely illustrated, in a mouse model, the large inter-individual variations in the height of the CMV-specific T cell responses to be dependent on the initial viral load which also influences the extent of memory T-cell inflation and phenotype of CMV-specific T cells. CMV infection generally leads to long-lasting substantial changes in circulating T cells, even more so in ESRD patients (13, 28–31). In particular, highly differentiated EMRA T cells lacking CD28 T cells are expanded in the CD8^+^ T cell population and to a lesser extent in the CD4^+^ T cell population (13–15). This typical footprint of CMV infection in the composition of circulating T cells was not found in the CMV-specific T cells of CMV-seronegative patients. This indicates that these patients have encountered CMV but only mounted a limited T cell response. The most likely explanation is low-level of exposure to viral antigens that may not result in the specific imprint induced by high viral antigen exposure which results in sustained CMV-specific IgG serum titers (32, 33). Furthermore, a proportion of CMV-seropositive patients, had no or low frequencies of CMV-specific CD8^+^ T cells but had CMV-specific CD4^+^ T cells as well as CMV-IgG-producing cells before transplantation, that were sufficient to prevent from a CMV reactivation. This was in contrast with a paper by Tey et al. that described hematopoietic stem cell transplant recipients, a different group of immunocompromised patients, deficient in reconstituting CMV-specific immunity to have higher viral loads (34). Our data are remarkably consistent with a recent study by Lucia et al. (11) that identified CMV-specific T cell responses in 30% of CMV seronegative individuals. However, their data were generated by ELISpot and therefore lack a more in-depth analysis of the T cell subsets involved. They also claim the detection of CMV-specific memory B cells but frequencies were unfortunately not reported.

In conclusion, assessment of anti-CMV-IgG does not seem to suffice for properly identifying CMV-naive individuals, as a significant proportion has a weak cellular CMV-specific response, which is associated with protection against CMV viremia after transplantation with a CMV-seropositive donor kidney. We therefore propose to include assessment of cellular immunity in the risk assessment before kidney transplantation. Furthermore, monitoring of CMV-specific cellular immunity following transplantation, i.e., at the end of anti-CMV prophylaxis might be useful for further identifying patients at risk for a CMV viremia, requiring additional antiviral therapy or adjustment of immunosuppressive medication (35).

## Ethics Statement

All patients gave written informed consent to participate in this study. The study was approved by the Medical Ethical Committee of the Erasmus MC (METC number 2010-080) and conducted in accordance with the Declaration of Helsinki and the Declaration of Istanbul.

## Author Contributions

NL designed the experiments, performed and analyzed the data, and wrote the manuscript; LH, BD, and RM performed the experiments and analyzed the data; JK interpreted the data and edited the manuscript; and MB designed the experiments and edited the manuscript.

## Conflict of Interest Statement

The authors declare that the research was conducted in the absence of any commercial or financial relationships that could be construed as a potential conflict of interest.

## Supplementary Material

Figure S1 Typical gating strategy for cytomegalovirus (CMV)-specific CD137-expressing and cytokine-producing CD4^+^ T cells. A typical flow-cytometric example is depicted for analysis of CMV-specific CD137-expressing and cytokine-producing CD4^+^ T cells for a CMV-seropositive **(A–C)** and CMV-seronegative **(D–F)** patient. Briefly, based on forward/sideward characteristics, live cells are gated and depicted in a dot plot to further select CD4^+^ and CD8^+^ T cells. The CMV-specific CD137-expressing CD4^+^ T cells are selected [**(A,D)**, first plot] as well as the IFNγ and IL-2 cells within CD137-expressing CD4^+^ T cells [**(A,D)**, second plot]. CD137-expressing CD4^+^ T cells are subsequently dissected into naive and different memory T cell subsets [**(A,D)**, third plot] using CCR7 and CD45RO expression [CCR7^+^CD45RO^−^: naive, CCR7^+^CD45RO^+^: central memory (CM), CCR7^−^CD45RO^+^: effector memory (EM) and CCR7^−^CD45RO^−^: EMRA] or CD28^+^ and CD28^null^ T cells [**(A,D)**, fourth plot]. IFNγ **(B,E)** and IL-2 **(C,F)**-producing CD137-expressing CD4^+^ T cells were analyzed in a similar way. Furthermore, the same approach was also applied for analysis of CMV-specific CD137-expressing and cytokine-producing CD8^+^ T cells (not shown). Percentages of CD137-expressing CD4^+^ T cells are of total CD4^+^ T cells and percentages of cytokine^+^ within CD137^+^CD4^+^ T cells are given as a proportion of CD137^+^CD4^+^ T cells (set to 100%) and in brackets the frequency of CD137^+^cytokine^+^ within total CD4^+^ T cells is depicted. The dissection with respect to a certain T-cell phenotype is done by setting the% of CD137^+^ or CD137^+^cytokine^+^CD4^+^ T cells to 100%.

Figure S2 CD137-expressing and cytokine-producing T cells upon stimulation with phorbol myristate acetate (PMA) and ionomycin. Peripheral mononuclear cells of patients were stimulated for 12-h in presence of brefeldin A and αCD49d alone or with a mixture of PMA and ionomycin. Subsequently, cells are cell surface and intracellular stained to determine the maximal capacity of T cells to express CD137 and produce cytokines. PMA/ionomycin-induced CD137-expressing CD4^+^ **(A)** and CD8^+^ **(B)** T cells, corrected for background (αCD49d only), are depicted as a percentage of total CD4^+^ or CD8^+^ T cells. A similar approach is followed for PMA/ionomycin-induced CD137-expressing IFN-γ- and IL-2-producing CD4^+^ **(C,E)** and CD137-expressing IFN-γCD8^+^ T cells **(D)**. Closed and open symbols/bars represent cytomegalovirus (CMV)-seronegative and CMV-seropositive patients, respectively.
