# Supplementary figures and images for: Protective Cytomegalovirus (CMV)-Specific T-Cell Immunity Is Frequent in Kidney Transplant Patients without Serum Anti-CMV Antibodies
Source: Front Immunol. 2017 Sep 12;8:1137. doi: 10.3389/fimmu.2017.01137 (PMC5600906; doi:10.3389/fimmu.2017.01137)

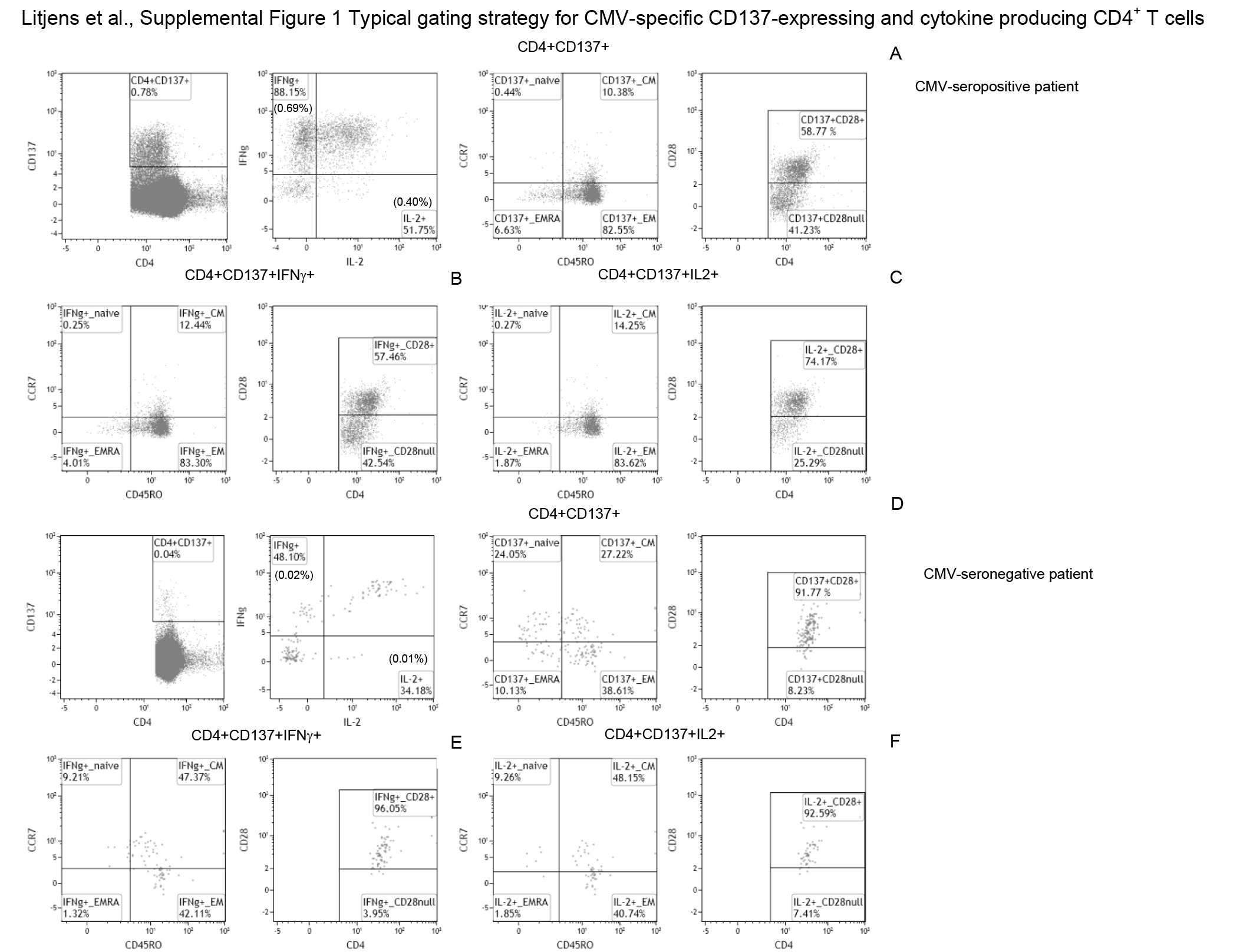

Supplement: Figure S1 — Typical gating strategy for cytomegalovirus (CMV)-specific CD137-expressing and cytokine-producing CD4+ T cells. A typical flow-cytometric example is depicted for analysis of CMV-specific CD137-expressing and cytokine-producing CD4+ T cells for a CMV-seropositive (A–C) and CMV-seronegative (D–F) patient. Briefly, based on forward/sideward characteristics, live cells are gated and depicted in a dot plot to further select CD4+ and CD8+ T cells. The CMV-specific CD137-expressing CD4+ T cells are selected [(A,D), first plot] as well as the IFNγ and IL-2 cells within CD137-expressing CD4+ T cells [(A,D), second plot]. CD137-expressing CD4+ T cells are subsequently dissected into naive and different memory T cell subsets [(A,D), third plot] using CCR7 and CD45RO expression [CCR7+CD45RO−: naive, CCR7+CD45RO+: central memory (CM), CCR7−CD45RO+: effector memory (EM) and CCR7−CD45RO−: EMRA] or CD28+ and CD28null T cells [(A,D), fourth plot]. IFNγ (B,E) and IL-2 (C,F)-producing CD137-expressing CD4+ T cells were analyzed in a similar way. Furthermore, the same approach was also applied for analysis of CMV-specific CD137-expressing and cytokine-producing CD8+ T cells (not shown). Percentages of CD137-expressing CD4+ T cells are of total CD4+ T cells and percentages of cytokine+ within CD137+CD4+ T cells are given as a proportion of CD137+CD4+ T cells (set to 100%) and in brackets the frequency of CD137+cytokine+ within total CD4+ T cells is depicted. The dissection with respect to a certain T-cell phenotype is done by setting the% of CD137+ or CD137+cytokine+CD4+ T cells to 100%. [file image_1.jpeg]

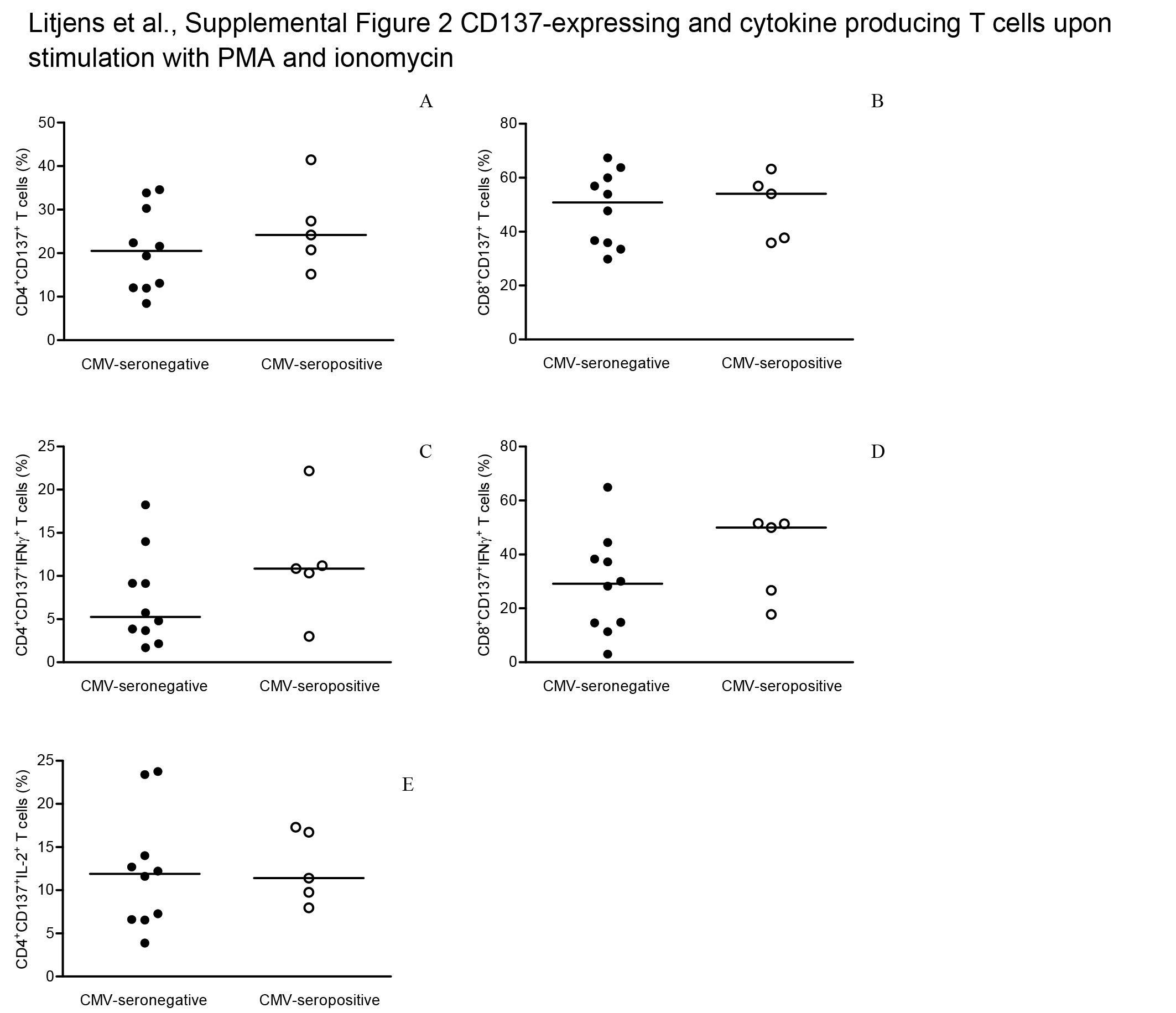

Supplement: Figure S2 — CD137-expressing and cytokine-producing T cells upon stimulation with phorbol myristate acetate (PMA) and ionomycin. Peripheral mononuclear cells of patients were stimulated for 12-h in presence of brefeldin A and αCD49d alone or with a mixture of PMA and ionomycin. Subsequently, cells are cell surface and intracellular stained to determine the maximal capacity of T cells to express CD137 and produce cytokines. PMA/ionomycin-induced CD137-expressing CD4+ (A) and CD8+ (B) T cells, corrected for background (αCD49d only), are depicted as a percentage of total CD4+ or CD8+ T cells. A similar approach is followed for PMA/ionomycin-induced CD137-expressing IFN-γ- and IL-2-producing CD4+ (C,E) and CD137-expressing IFN-γCD8+ T cells (D). Closed and open symbols/bars represent cytomegalovirus (CMV)-seronegative and CMV-seropositive patients, respectively. [file image_2.jpeg]
